# Surface Nanoengineering of Gold via Oxalic Acid Anodization: Morphology, Composition, Electronic Properties, and Corrosion Resistance in Artificial Saliva

**DOI:** 10.3390/ma19020335

**Published:** 2026-01-14

**Authors:** Bożena Łosiewicz, Delfina Nowińska, Julian Kubisztal, Patrycja Osak

**Affiliations:** Faculty of Science and Technology, Institute of Materials Engineering, University of Silesia in Katowice, 75 Pułku Piechoty 1A, 41-500 Chorzów, Poland

**Keywords:** nanoporous gold, gold anodization, oxalic acid, surface nanoengineering, contact potential difference, corrosion resistance, artificial saliva, biomedical materials

## Abstract

**Highlights:**

**What are the main findings?**

- Oxalic acid anodization enables controlled formation of nanoporous gold layers.
- Increasing acid concentration increases pore size, porosity, and layer thickness.
- Nanoporous gold shows altered electronic properties and work function.

**What are the implications of the main findings?**

- Moderate anodization (0.3 M oxalic acid) offers optimal structural uniformity.
- Electronic and corrosion properties are tunable via anodization conditions.
- Nanoporous gold is promising for dental and biomedical surface applications.

**Abstract:**

Nanoporous gold (np-Au) has attracted significant attention for biomedical and electrochemical applications due to its high surface area, tunable morphology, and excellent biocompatibility. In this study, polycrystalline gold surfaces were modified by anodization in 0.3–0.9 M oxalic acid to produce np-Au layers. The influence of anodization conditions on surface morphology, chemical composition, electronic properties, and corrosion resistance in artificial saliva was systematically investigated. Surface morphology and porosity were analyzed by scanning electron microscopy combined with image analysis, revealing a transition from fine and uniform porosity to highly developed but structurally heterogeneous nanoporous structures with increasing oxalic acid concentration. Energy-dispersive spectroscopy confirmed surface oxidation and adsorption of oxygen- and carbon-containing species after anodization, while gold remained the dominant component. Scanning Kelvin probe measurements demonstrated significant modifications of surface electronic properties, including changes in contact potential difference, governed by nanostructure geometry and surface chemistry. Electrochemical tests in artificial saliva showed that increasing nanoporousness led to reduced thermodynamic stability, with the sample anodized in 0.3 M oxalic acid providing the most favorable balance between corrosion resistance and surface activity. These results demonstrate that oxalic acid anodization is a simple and effective approach for tailoring gold surfaces for biomedical applications, particularly in dentistry.

## 1. Introduction

Polycrystalline gold (p-Au) is a widely used material in surface engineering, biomaterials, and electrochemical devices due to its excellent electrical conductivity, corrosion resistance, and biocompatibility [1]. Advances in nanotechnology have enabled controlled modification of p-Au at the nanoscale, leading to the development of nanoporous gold (np-Au) with a significantly increased specific surface area. This enhances electrochemical and adsorption performance and expands applications in sensors, catalysis, and biomaterials, including promising uses in modern dentistry as implant coatings, drug carriers, and tissue scaffolds [1,2,3,4,5]. A crucial feature of np-Au is the ability to tailor its structure to specific functional requirements, as demonstrated in recent studies on flexible electrodes and tissue engineering applications [6,7].

The most commonly employed method for np-Au fabrication is dealloying, which involves selective removal of less noble metals from Au alloys, resulting in a porous gold network with a high surface-to-volume ratio [1,8,9,10,11,12,13,14,15,16,17]. Although this method allows control over porosity and layer thickness, it often produces irregular pore structures, may compromise mechanical stability, and poses challenges related to scalability, cost, and potential toxicity from residual chemicals [16,17].

Template-assisted synthesis (TAS) represents another approach, enabling precise control over pore size, topology, and layer thickness through the use of biocompatible templates such as polymers or biological structures [18,19,20,21,22,23]. Despite its advantages for biofunctionalization and three-dimensional nanostructure design, TAS is limited by high process complexity, cost, and the risk of defects or contamination during template removal [24,25,26].

Chemical reductive synthesis offers a more versatile and mild route to np-Au fabrication, based on the reduction of Au^3+^ ions to metallic gold and subsequent formation of porous three-dimensional networks [27,28,29,30]. Oxalic acid is frequently used as a reducing agent and growth regulator, enabling partial control over nanostructure morphology under relatively benign conditions, which is advantageous for biomaterial applications [27,28,29,30]. However, control over pore regularity and mechanical stability remains limited.

Lithography-based techniques, such as electron-beam and nanoimprint lithography, allow precise spatial control of np-Au architectures and integration with micro- and nanoelectronic systems, but their high cost, complexity, and low throughput restrict large-scale applications [2,31,32,33,34]. Self-assembly and nanoparticle-based approaches provide relatively fast and homogeneous routes for the formation of gold-based nanostructures, although with limited control over long-range order and mechanical stability [35,36,37].

In contrast to the methods for obtaining np-Au presented above, which are based mainly on reduction and dealloying processes, anodization of gold in oxalic acid solutions represents an interesting electrochemical alternative for producing this type of surface [28,38]. Anodization enables the formation of a np-Au layer through the generation of a porous gold oxide on the surface, which in a subsequent step is partially or completely reduced to nanoporous gold [39,40]. During the anodization, characteristic dark layers (so-called black gold) with high porosity, increased roughness, and enlarged electrochemically active surface area are formed [4,28], which is particularly advantageous in the context of osteointegration of metallic biomaterials [41]. By controlling parameters such as electrolyte composition, process duration, and current–time characteristics, it is possible to tailor the degree of porosity, pore size, and thickness of the resulting layer [28]. Oxalic acid is most commonly used in the anodization, as it promotes the formation of a regular and stable porous structure [38,42]. It is a weak acid and is characterized by relatively mild chemical activity. As an organic acid, it forms stable oxalate complexes with gold, enabling uniform dissolution and reconstruction of the gold surface layer during anodization [28]. Structures formed in oxalic acid solutions develop in a controlled manner without abrupt degradation of the surface layer, which is more difficult to achieve in strongly aggressive inorganic electrolytes such as H_2_SO_4_ or HClO_4_ [4]. In addition, oxalic acid provides good ionic conductivity of the solution, which contributes to the stability of the applied voltage and the reproducibility of the process [28]. As a result, anodization of gold in oxalic acid allows precise control over surface morphology and enables the fabrication of np-Au with high chemical purity and excellent biocompatibility, without introducing toxic ions such as chlorides or nitrates into the surface structure [38,39]. Anodization is a relatively simple and scalable method that enables the formation of nanoporous structures without the need for sophisticated equipment.

Despite increasing interest in np-Au, significant knowledge gaps remain in the available literature. The effects of anodization in oxalic acid on polycrystalline gold are insufficiently explored compared to dealloying and TAS methods [43]. Comprehensive analyses correlating surface morphology, chemical composition, and electronic properties of np-Au with its corrosion resistance in biological environments, such as artificial saliva, are still lacking, in analogy to studies conducted for commercially used titanium [44]. Furthermore, limited attention has been paid to the influence of anodization parameters on the final surface properties of p-Au, hindering the rational design of biofunctional surfaces [45].

Therefore, the aim of this study is a comprehensive characterization of p-Au surfaces anodized in oxalic acid, focusing on morphology, chemical composition, electronic properties, and corrosion resistance in artificial saliva, to fill the existing gaps in the literature and to support the development of functional gold surfaces for biomedical applications.

## 2. Materials and Methods

### 2.1. Preparation of p-Au Electrodes

Gold wire with a diameter of 0.5 mm and a purity of 99.99 wt.% was supplied by Goodfellow Cambridge Ltd. (Huntingdon, UK). Prior to each electrochemical measurement, the exposed wire surface was mechanically treated to remove organic and oxide contaminants. Grinding was carried out sequentially using silicon carbide abrasive papers with grit sizes ranging from 1200 to 4000, followed by polishing on a felt pad with Al_2_O_3_ suspensions (0.3 µm and 0.05 µm; Struers Inc., Cleveland, OH, USA) until a mirror-like finish was obtained. Surface finishing was performed using a Metkon Forcipol 102 metallographic grinding and polishing machine (Metkon Instruments Inc., Bursa, Turkey) operated at 250 rpm. After polishing, the wire was thoroughly rinsed with ultrapure water (Milli-Q^®^ Advantage A10 Water Purification System, Millipore SAS, Molsheim, France) and subsequently sonicated in an ultrasonic cleaner (USC 300 TH, VWR International, Radnor, PA, USA) for 5 min in ethanol (Avantor Performance Materials Poland S.A., Gliwice, Poland), followed by 5 min in ultrapure water, to remove residual abrasive particles and organic contaminants. After each step, the sample was rinsed with a fresh portion of the corresponding solvent. To ensure complete removal of organic residues, the wire was immersed for 1–2 min in a freshly prepared piranha solution (H_2_SO_4_:H_2_O_2_ = 3:1, *v*/*v*) and then immediately rinsed thoroughly with ultrapure water.

In order to obtain a reproducible and electrochemically active p-Au surface, each electrode was electrochemically activated immediately prior to anodization by multiple cyclic voltammetry (CV) scans. The CVs were recorded in 0.5 M H_2_SO_4_ electrolyte over a potential range from −0.2 to 1.5 V vs. Hg|Hg_2_Cl_2_|KCl_(sat.)_ electrode, using a scan rate of 50–100 mV s^−1^. The number of cycles ranged from 10 to 20, until stable and reproducible CV curves were obtained, using a modular high-current potentiostat–galvanostat electrochemical system (Metrohm Autolab B.V., Utrecht, The Netherlands). After the cleaning procedure, the working electrode was dried under a nitrogen stream and immediately used for electrochemical measurements. The geometric surface area of the gold wire working electrode was A = 0.159 cm^2^. A platinum foil with a geometric surface area of 8 cm^2^ was used as the counter electrode.

### 2.2. Electrochemical Production of np-Au Electrodes

The anodization of p-Au electrodes was carried out in oxalic acid solutions with concentrations of 0.3 M, 0.6 M, and 0.9 M. The samples of p-Au were used as the anodic material. The anodization process was performed in a conventional two-electrode configuration using a PWR800H high-current power supply (Kikusui Electronics Corp., Yokohama, Japan). A platinum foil served as the cathode. The distance between the electrodes was kept constant for all experiments and was 1.5 cm. Electrolyte solutions were prepared immediately before the measurements using ultrapure water (resistivity 18.2 MΩ × cm). Anodization was conducted at a constant voltage of 5 V for 20 min at room temperature (~22 °C), without additional stirring of the electrolyte. During the process, the current–time response was monitored to evaluate the kinetics of electrochemical oxidation of p-Au. After anodization, np-Au electrodes were immediately removed from the electrolyte, thoroughly rinsed with ultrapure water to remove residual oxalic acid, and then dried under a stream of nitrogen. The obtained anodized surfaces were stored in a dry environment for further structural and electrochemical analyses.

The selected anodization parameters were additionally validated during preliminary trials at different voltages and times, which confirmed their suitability for producing stable np-Au layers. The present work focuses exclusively on the influence of oxalic acid concentration, while a detailed optimization of anodization parameters is beyond its scope.

### 2.3. Scanning Electron Microscopy

Surface morphology and thickness of np-Au layers formed during the anodization process were examined using a JEOL JSM-6480 scanning electron microscope (SEM, Tokyo, Japan). Measurements were performed at an accelerating voltage in the range of 10–20 kV, depending on the required surface sensitivity and image resolution, with a working distance of approximately 10 mm. Secondary electron (SE) imaging mode was primarily used to reveal surface topography, while cross-sectional observations were employed to assess the thickness of the np-Au layers. All samples were examined without additional conductive coatings to preserve the native surface state and avoid contributions from foreign materials. Prior to SEM analysis, the samples were gently cleaned with ethanol and dried under ambient conditions.

To minimize beam-induced charging and contamination effects, imaging parameters were carefully optimized, particularly for the highly porous anodized samples. The obtained SEM micrographs were used for qualitative morphological assessment and provided the basis for subsequent quantitative image analysis.

### 2.4. Image Analysis and Porosity Evaluation of np-Au

Quantitative analysis of surface porosity was performed using ImageJ software (version 1.54p, National Institutes of Health, Bethesda, MD, USA) based on high-resolution SEM micrographs [46]. Prior to analysis, SEM images were converted to 8-bit grayscale and calibrated using the scale bar provided in each micrograph. Image preprocessing included noise reduction by Gaussian filtering to improve contrast between the nanoporous features and the solid gold matrix.

Nanopores were segmented using a local thresholding approach, and the resulting binary images were further processed by morphological operations (opening and hole filling) to remove isolated noise and to improve pore definition. In cases where adjacent pores were in contact, a watershed-based separation procedure was applied. Porosity was quantified as the area fraction of nanopores relative to the total analyzed surface area. Particle analysis was carried out using the Analyze Particles function in ImageJ, allowing determination of pore size distribution, pore area, and number density. Only pores within a predefined size range were considered in order to exclude artifacts arising from image noise or surface defects. To ensure statistical reliability, the analysis was performed on multiple SEM images acquired from different regions of each sample, and the reported values represent the average results. The porosity values obtained from ImageJ analysis should be regarded as two-dimensional surface porosity estimates derived from SEM images.

### 2.5. Energy-Dispersive Spectroscopy

Energy-dispersive spectroscopy (EDS) analysis was conducted using a JEOL JSM-6480 SEM equipped with an energy-dispersive X-ray detector. Measurements were performed at an accelerating voltage of 15 kV and a working distance of approximately 10 mm. The beam current was adjusted to obtain sufficient count statistics while minimizing beam-induced contamination. EDS spectra were acquired from several randomly selected areas of each sample to ensure representativeness. Quantitative elemental analysis was carried out using a standardless ZAF correction method, and the results were reported in terms of atomic (at.%) and weight (wt.%) percentages. No additional conductive coating was applied prior to analysis in order to avoid contributions from foreign elements.

For anodized samples, particular attention was paid to the detection of low-energy X-ray lines corresponding to carbon (C Kα, ~0.28 keV) and oxygen (O Kα, ~0.53 keV). Due to the highly porous surface morphology and large specific surface area of np-Au layers, the obtained EDS data should be regarded as semi-quantitative, especially with respect to light elements.

### 2.6. Contact Potential Difference

Contact potential difference (CPD) measurements were performed using a scanning Kelvin probe (SKP) (U-SKP-150, Uniscan Instruments, Buxton, UK), which enables non-contact mapping of the local electronic properties of surfaces. The technique is based on measuring the compensating voltage required to nullify the capacitive current generated by the periodic modulation of the distance between a vibrating reference probe and the sample surface. The recorded CPD values correspond to local differences in work function between the Kelvin probe and the investigated surface. All measurements were carried out under ambient laboratory conditions at room temperature, without humidity control. A metallic Kelvin probe with a stable and well-defined work function was used and calibrated against a reference Au substrate prior to the measurement series. CPD maps were acquired over surface areas of 2 × 2 mm^2^, allowing assessment of the macroscopic uniformity of the electrostatic potential across the nanoporous gold surfaces. The electronic characteristics of the investigated materials were examined using a PAR Model 370 scanning electrochemical workstation (Princeton Applied Research, Oak Ridge, TN, USA), equipped with an integrated SKP370 module and an optical video microscope (VCAM3).

CPD measurements were conducted for np-Au samples produced by anodizing in oxalic acid solutions with concentrations of 0.3 M, 0.6 M, and 0.9 M. For each sample, several randomly selected regions were analyzed to ensure representative sampling of the surface. Statistical analysis of CPD distributions was performed by constructing histograms from the full CPD maps after removal of edge artifacts and suppression of measurement noise. A smooth p-Au substrate was used as a reference sample, enabling direct comparison of CPD values and distributions before and after anodizing. No additional surface treatment was applied after the electrochemical process, allowing evaluation of the intrinsic effect of anodizing conditions on the electronic properties of the np-Au surfaces.

### 2.7. Corrosion Resistance in Artificial Saliva

The corrosion resistance of p-Au and np-Au electrodes against electrochemical corrosion was investigated in deaerated artificial saliva with a pH of 7.4 ± 0.1 at a temperature of 36.6 °C (Table 1).

The pH of the solutions was adjusted using a 4% NaOH solution and a 1% C_3_H_6_O_3_ solution. Ultrapure water was used for the preparation of all solutions.

Electrochemical corrosion tests were carried out in a conventional three-electrode setup, where the examined electrode served as the working electrode, a platinum foil acted as the counter electrode, and a saturated calomel electrode was used as the reference electrode, introduced into the electrolyte via a Luggin capillary. To prevent re-oxygenation of the electrolyte, a continuous argon atmosphere was maintained above the solution surface. All measurements were performed using an Autolab/PGSTAT12 (Electrochemical data were collected and analyzed with GPES for Windows (v4.9)) potentiostat/galvanostat controlled by a computer (Metrohm Autolab B.V., Utrecht, The Netherlands).

The open-circuit potential (E_OC_) was stabilized for 2 h. Anodic polarization curves were recorded starting from a potential 150 mV below the stabilized E_OC_, at a scan rate of 1 mV s^−1^, up to a final potential of 0.57 V.

## 3. Results

### 3.1. The Effect of Anodizing on the Surface Morphology and Porosity of p-Au

**Figure 1:**
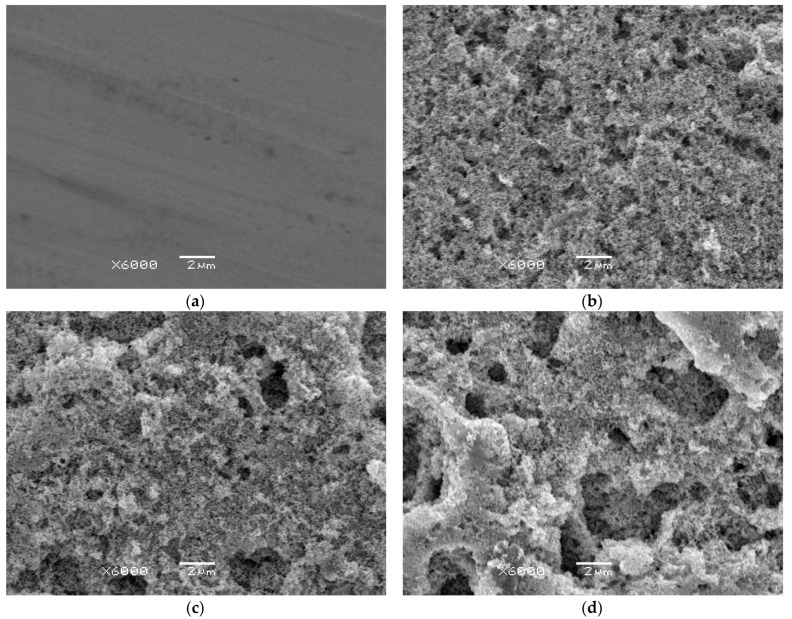
SEM image of the gold microstructure at ×6000 magnification: (**a**) polycrystalline gold (p-Au) before anodization; (**b**) nanoporous gold (np-Au) after anodization in an oxalic acid solution with a concentration of 0.3 M; (**c**) np-Au after anodization in an oxalic acid solution with a concentration of 0.6 M; (**d**) np-Au after anodization in an oxalic acid solution with a concentration of 0.9 M.

To assess the influence of oxalic acid concentration on the surface morphology of p-Au, a comparative analysis of SEM images was performed. Observations were carried out for the p-Au substrate prior to anodization (Figure 1a) as well as for np-Au layers formed after anodization in oxalic acid solutions with concentrations of 0.3 M (Figure 1b), 0.6 M (Figure 1c), and 0.9 M (Figure 1d). The analysis aimed to demonstrate changes in surface topography and to determine the degree of development of the np-Au structure as a function of electrolyte concentration.

SEM image analysis demonstrates a significant influence of oxalic acid concentration on the morphology of porous gold obtained by anodization of an Au substrate. The base material prior to anodization is characterized by a relatively smooth and compact surface with clearly visible grain boundaries observed as dark bands, which is typical for p-Au (Figure 1a). At the analyzed micrometric scale, subtle, parallel, and oriented scratches resulting from mechanical processing are also noticeable, while the absence of pronounced surface defects indicates a low degree of surface roughness of the investigated material.

As a result of anodization in a 0.3 M oxalic acid solution, a distinct change in surface morphology is observed (Figure 1b) compared with the initial material. The surface adopts a fine-grained structure with slightly increased roughness and the onset of non-uniform nanoporous features. The formed structure remains relatively compact, and the observed pores are small and poorly developed, indicating a moderate intensity of the anodization process and a limited extent of electrochemical dissolution of the gold surface layer. This suggests that at low electrolyte concentration the anodization process proceeds in a controlled manner and promotes the initiation of porosity without its full development.

Anodization in a 0.6 M oxalic acid solution leads to a pronounced development of the porous gold structure (Figure 1c) compared with the sample anodized at a lower electrolyte concentration. SEM images indicate a significant increase in surface roughness, as well as the presence of more numerous and more clearly defined pores and greater topographical heterogeneity. Local areas of deepened morphology and irregular depressions are observed, suggesting an intensification of electrochemical etching processes and a stronger influence of grain boundaries on the anodization behavior. As a result, the surface becomes more morphologically developed, evidencing the progressive formation of a porous gold structure. Compared with the sample anodized in a 0.3 M oxalic acid solution, a transition from the initiation of porosity to its distinct development is observed.

In contrast, anodization in a 0.9 M oxalic acid solution results in the most advanced reconstruction of the gold surface morphology among the analyzed conditions (Figure 1d). SEM images reveal the presence of large, irregular pores and caverns, as well as local structural collapse, leading to a highly diversified and heterogeneous surface topography. Compared with lower electrolyte concentrations, a significant loss of structural uniformity of the porous layer is observed, and the anodization process shifts toward intensive and aggressive surface dissolution, which may lead to partial destruction of the anodic layer. This indicates that the optimal anodization conditions favoring controlled formation of an ordered porous gold structure have been exceeded. A comparison of the SEM results clearly shows that increasing oxalic acid concentration leads from the initiation of porosity (0.3 M), through its development (0.6 M), to the dominance of destructive surface dissolution processes (0.9 M).

It should be noted that the present study is focused on surface-related and functional properties of anodized gold rather than on the analysis of bulk crystalline structure. Under the anodization conditions applied, gold does not form stable, bulk crystalline oxide phases [1]. Therefore, SEM combined with quantitative image analysis provides sufficient information on surface morphology and porosity at the length scales relevant for electrochemical and biomedical applications, while techniques such as XRD or TEM were not essential within the scope of this work.

**Figure 2:**
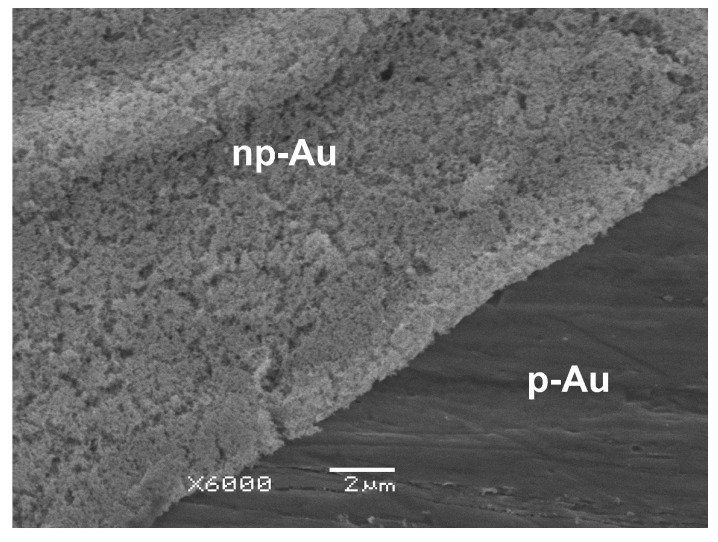
SEM cross-sectional image at ×6000 magnification showing the nanoporous gold (np-Au) layer formed by anodization in a 0.3 M oxalic acid solution and the underlying polycrystalline gold (p-Au) substrate.

Complementing the surface morphology analysis are cross-sectional SEM images, which enable evaluation of the extent and continuity of the porous layer formed as a result of anodization (Figure 2).

The representative cross-sectional SEM image allows evaluation of the structure of the np-Au layer formed as a result of anodization in a 0.3 M oxalic acid solution with respect to the p-Au substrate. A distinct boundary between the smooth, unmodified base material and the developed, porous surface layer with a sponge-like character is clearly visible. The porous structure exhibits a high degree of development and continuity over the entire observed cross-sectional length, indicating a uniform anodization process. The absence of visible cracks or delamination at the layer–substrate interface suggests good adhesion of the np-Au layer to the base material. The obtained image confirms that anodization leads to a significant modification of the surface topography through the formation of a clearly defined porous layer.

The thickness of the np-Au layer increases systematically with increasing oxalic acid concentration. For the sample anodized in a 0.3 M H_2_C_2_O_4_ solution, the np-Au layer reaches a thickness of 0.84 ± 0.23 µm. Increasing the electrolyte concentration to 0.6 M results in an approximate doubling of the layer thickness to about 1.68 ± 0.46 µm, while further increasing the concentration to 0.9 M leads to a continued growth of the porous layer, reaching approximately 2.52 ± 0.69 µm. This trend indicates that higher oxalic acid concentrations promote more intense anodic dissolution and a higher rate of oxide formation, which in turn favor the development of thicker np-Au layers.

**Figure 3:**
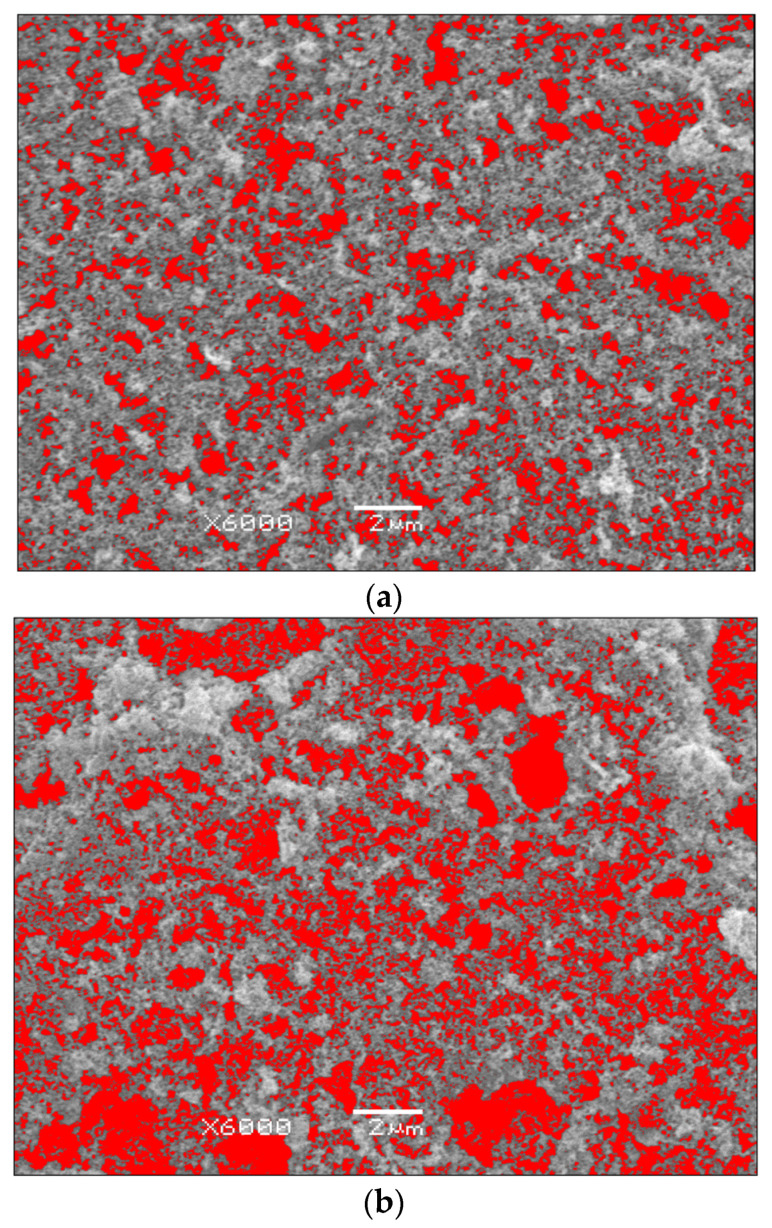

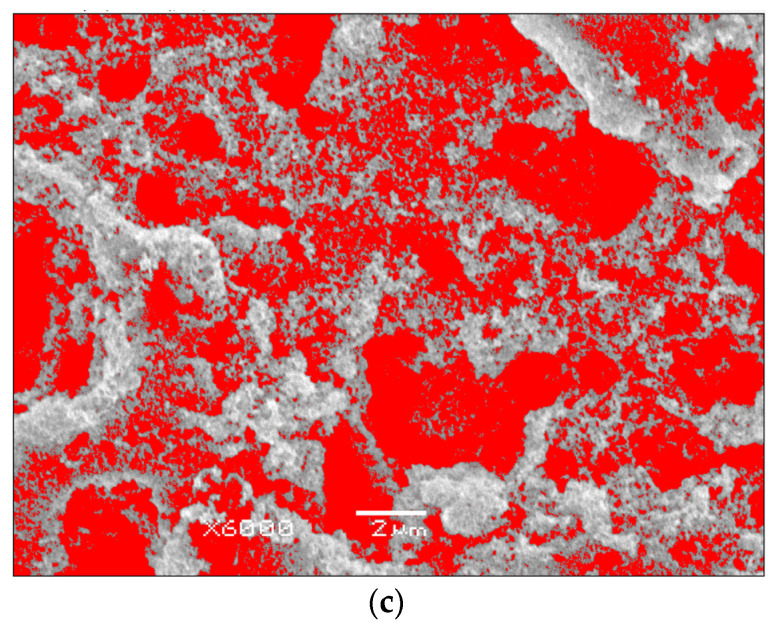
Grayscale SEM images with nanopores highlighted in red (ImageJ analysis), acquired at ×6000 magnification for nanoporous gold (np-Au) surfaces formed by anodization in oxalic acid solutions: (**a**) 0.3 M; (**b**) 0.6 M; (**c**) 0.9 M.

For a quantitative assessment of the porous gold structure, SEM images presented in Figure 1 were analyzed using ImageJ software. Grayscale SEM images with nanopores highlighted in red were used to determine pore geometric parameters and surface porosity (Figure 3). The analysis aimed at quantitatively comparing the effect of oxalic acid concentration during anodization on the development of porous gold (Table 2).

The quantitative analysis of the surface porosity of np-Au reveals a clear dependence between the oxalic acid concentration and the geometric parameters of the nanopores (Table 2). With increasing electrolyte concentration, a systematic increase in both the mean and median nanopore diameter is observed, indicating an intensification of dissolution processes. At the same time, a significant increase in the standard deviation can be noted, particularly for the sample anodized in a 0.9 M oxalic acid solution, which reflects the growing heterogeneity in the nanopore size distribution. An opposite trend is observed for pore density, which decreases with increasing electrolyte concentration, suggesting a transition from a structure composed of numerous small pores to a morphology characterized by a smaller number of larger and more irregular pores. Concurrently, the surface porosity increases from 18% for the sample anodized in a 0.3 M oxalic acid solution to 48% at the highest analyzed concentration, confirming the progressive development of the porous gold structure with increasing aggressiveness of the anodization conditions.

The effective specific surface area (SSA) of np-Au was estimated using a simplified geometric model based on SEM-derived pore diameter and surface porosity values. Assuming an open-cell porous structure with cylindrical pores, the specific surface area was calculated according to SSA = 4φ/(d·ρ), where φ is surface porosity, d is average pore diameter, and ρ is the density of gold. The estimated values (Table 2) reveal that despite a continuous increase in surface porosity with increasing oxalic acid concentration, the effective SSA does not increase monotonically. The highest SSA values are obtained for samples anodized in 0.3 M and 0.6 M oxalic acid, whereas further increase in porosity at 0.9 M is accompanied by pore coarsening and a reduction in SSA. This result indicates that surface coarsening dominates over porosity increase at high electrolyte concentrations, leading to a lower surface-to-volume ratio.

### 3.2. The Effect of Anodizing on the Chemical Composition of the p-Au Surface

**Figure 4:**
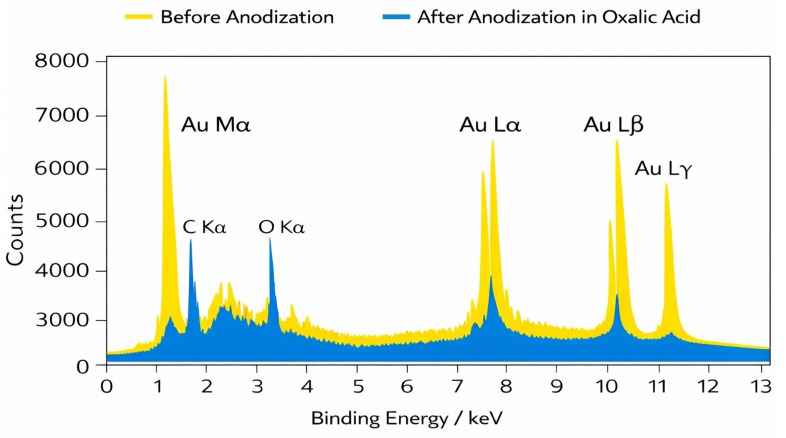
EDS spectra of gold samples before anodization and after anodization in oxalic acid. Characteristic gold peaks (Au Mα, Au Lα, Au Lβ, and Au Lγ) as well as signals from light elements (C Kα and O Kα) are observed. After anodization, noticeable changes in peak intensities and an increased oxygen contribution are detected, indicating the formation of an oxide layer on the sample surface.

In the EDS spectrum of the sample before anodization (p-Au), intense peaks characteristic of gold are dominant, particularly the Au Mα line and the Au L-series lines (Au Lα, Au Lβ, and Au Lγ), confirming the high gold content of the material (Figure 4). The low intensity of oxygen and carbon signals suggests a relatively clean surface with only minor surface contamination.

After anodization in oxalic acid, a clear increase in the intensity of the O Kα peak is observed, indicating the presence of oxygen on the sample surface and confirming the formation of an oxide layer. At the same time, the relative intensity of the gold peaks decreases, which can be attributed to attenuation of the Au signal by the newly formed oxide layer and changes in X-ray generation conditions in the near-surface region. The presence of the C Kα signal in the post-anodization spectrum may result from adsorption of organic contaminants, electrolyte residues, or exposure of the sample to the ambient atmosphere. The absence of additional peaks from other elements indicates that the anodization process did not introduce significant metallic impurities, and the observed spectral changes are primarily related to chemical and structural modifications of the surface layer. The EDS spectra confirm the effectiveness of the anodization process and its significant influence on the chemical composition of the near-surface region of gold.

Quantitative EDS analysis shows that polycrystalline gold prior to anodization is composed almost entirely of metallic gold, with an atomic concentration of approximately 98.5–99.2 at.% Au. Minor contributions from carbon (0.5–1.0 at.%) and oxygen (≤0.5 at.%) are detected and can be attributed to surface contamination and adsorption from ambient air. The absence of detectable oxide phases and the clear dominance of gold in both atomic and weight percentages indicate a chemically clean and metallic surface state before anodization.

After anodization in oxalic acid, a substantial increase in the surface concentrations of carbon and oxygen is observed, accompanied by a corresponding decrease in the atomic fraction of gold to approximately 80–88 at.%. Oxygen content rises to about 6–10 at.% and carbon to approximately 6–12 at.%, which is attributed to the presence of oxygen-containing functional groups, surface adsorbates, and residual oxalate-derived species. Despite the reduced atomic fraction, gold remains the dominant component in terms of weight percentage (approximately 93–97 wt.%), confirming that the anodization process does not lead to the formation of bulk gold oxides and that metallic gold remains the primary constituent of the material. It should be emphasized that EDS is a semi-quantitative, surface-sensitive technique, and the measured carbon and oxygen contents are likely overestimated relative to the true bulk composition. Consequently, the detected increases in carbon and oxygen primarily reflect modifications of the near-surface region rather than changes in the bulk material.

Previous studies have demonstrated that gold does not form a stable bulk oxide under anodic conditions, and oxygen detected by surface-sensitive techniques is primarily associated with chemisorbed oxygen species or surface complexes rather than Au_2_O_3_ formation [48,49]. In np-Au, the high surface area strongly enhances adsorption of carbonaceous and oxygen-containing species originating from electrolytes or ambient exposure, which is frequently reflected in EDS and XPS measurements [34,49,50]. Such surface chemistry, combined with nanoscale porosity, plays a crucial role in the optical response of so-called “black gold” materials [51].

It should be emphasized that EDS is a semi-quantitative technique and does not allow unambiguous identification of the chemical states of oxygen or carbon. Accordingly, the EDS results presented here are interpreted exclusively as qualitative confirmation of the presence of oxygen- and carbon-containing species in the near-surface region of anodized gold. The chemical nature of the anodized gold surface, including oxygen adsorption as well as the possible presence of oxalate- and hydroxyl-related species, is discussed on the basis of previously reported XPS studies, without overinterpretation of the EDS data [1,43,48,49].

### 3.3. The Effect of Anodizing on the Electronic Properties of p-Au

**Figure 5:**
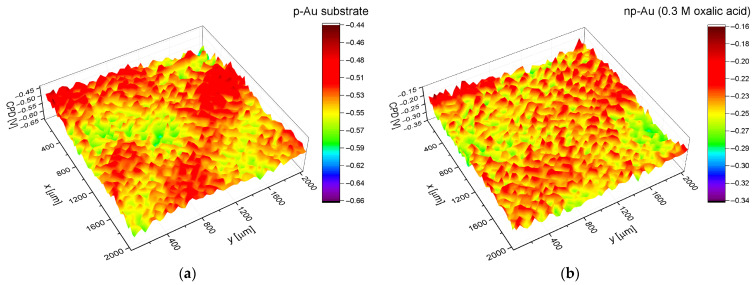

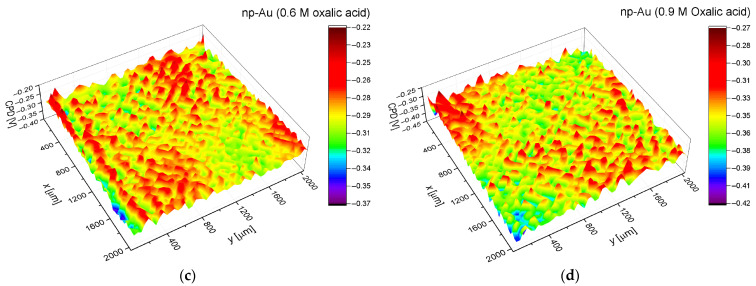
The CPD distribution map on the surface of gold: (**a**) polycrystalline gold (p-Au) before anodization; (**b**) nanoporous gold (np-Au) after anodization in an oxalic acid solution with a concentration of 0.3 M; (**c**) np-Au after anodization in an oxalic acid solution with a concentration of 0.6 M; (**d**) np-Au after anodization in an oxalic acid solution with a concentration of 0.9 M.

Contact potential maps (CPD) obtained by the SKP method allowed for a detailed analysis of changes in the electronic properties of the p-Au surface as a result of anodization in an oxalic acid environment of varying concentrations (Figure 5). Comparison of p-Au with anodically produced np-Au clearly indicates that the anodization leads to a fundamental reorganization of both the morphology and local electronic properties of the surface.

For p-Au, the CPD contrast observed in Figure 5a is typical of polycrystalline metals and arises mainly from the presence of grains with different crystallographic orientations, which are characterized by different work functions [52]. The relatively narrow CPD distribution and the absence of sharp local anomalies suggest chemical homogeneity of the surface, with crystallographic anisotropy of gold remaining the dominant factor determining the CPD.

Anodization leads to a complete change in the character of the CPD maps (Figure 5b–d). For all np-Au samples, the grain-related contrast disappears, indicating that the electronic properties of the surface are no longer controlled by crystallographic orientation but instead are governed by the nanostructure and surface chemistry [53]. This is a direct consequence of the anodic formation of a network of nanopores and ligaments, which introduces a large number of surface defects, edges, and regions of high curvature [1].

The sample anodized in 0.3 M H_2_C_2_O_4_ exhibits the most homogeneous CPD distribution and a shift of the mean CPD value toward less negative potentials compared with p-Au (Figure 5b). This effect can be attributed to the formation of a relatively regular nanoporous structure with larger ligaments and a lower density of defects, leading to partial averaging of local electronic properties [29].

With increasing oxalic acid concentration to 0.6 M, a pronounced increase in local CPD fluctuations is observed (Figure 5c). More intensive anodization leads to a finer nanostructure and an increased number of edges, junctions, and areas with high surface energy, which enhances the spatial heterogeneity of the CPD [1]. In addition, the higher concentration of oxalate ions promotes their adsorption on the Au surface, resulting in the formation of surface dipoles and further modification of the local potential [54].

The most pronounced effects are observed for np-Au anodized in 0.9 M H_2_C_2_O_4_ (Figure 5d). This sample is characterized by the broadest CPD range and the most negative mean values in the entire series, reflecting an extremely developed and highly heterogeneous nanostructure. Under these conditions, the electronic properties of the surface are governed by a combination of very fine ligaments, a high density of defects, and strong chemical effects, including possible partial positive charging of surface Au atoms (Au^δ+^), which increases the effective work function [1,55].

Analysis of the entire sample series reveals a nonlinear dependence of CPD on the concentration of the anodizing electrolyte. Moderate anodization (0.3 M) leads to surface homogenization and a reduction of the effective work function, whereas further increases in electrolyte concentration (0.6–0.9 M) result in enhanced contact potential heterogeneity and a renewed increase in CPD [1,29]. These results demonstrate that the electronic properties of anodically produced nanoporous gold are determined by a subtle balance between nanostructure geometry, defect density, and surface chemistry [1].

**Figure 6:**
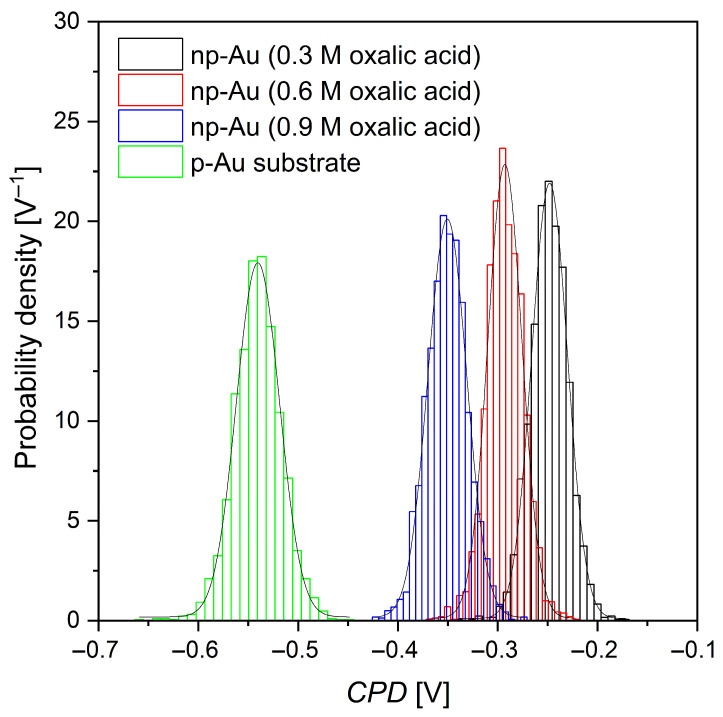
CPD distribution histogram of gold based on the CPD map shown in Figure 5, with a Gaussian fitting curve superimposed.

Based on the CPD distribution map of the gold surface shown in Figure 5, in which CPD is defined as the z-axis variable, the CPD values were extracted to produce the distribution histogram presented in Figure 6. CPD distributions obtained from histograms quantitatively confirm the qualitative observations derived from SKP maps and clearly indicate a significant influence of oxalic acid concentration during anodization on the electronic properties of the Au surface. Compared to the p-Au substrate, which is characterized by a narrow, unimodal CPD distribution mainly associated with the crystallographic orientation of the grains [52], anodized np-Au samples exhibit a systematic shift of the CPD distributions toward less negative values accompanied by an increase in their width. For the sample anodized in 0.3 M H_2_C_2_O_4_, the most homogeneous CPD distribution is observed, indicating a relatively regular nanostructure and a limited number of local surface defects. Increasing the electrolyte concentration to 0.6 M leads to a pronounced broadening of the histogram and a shift of the maximum toward more negative CPD values, reflecting an increase in the electronic inhomogeneity of the surface. The most pronounced effect is observed for np-Au anodized in 0.9 M H_2_C_2_O_4_, where a broad CPD distribution and the presence of a tail toward low potential values indicate the coexistence of regions with strongly varying local work functions. This effect can be attributed to extensive surface development and a high density of defects induced by intense anodization, which is consistent with previously described mechanisms of porous Au nanostructure formation in oxalic acid solutions [56].

The statistical parameters of the CPD, namely the arithmetic mean (CPD_av_), root mean square (CPD_q_), skewness (CPD_sk_), and excess kurtosis (CPD_ku_), obtained from the CPD distribution map (Figure 5) and histogram (Figure 6), are listed in Table 3.

Anodization of a gold surface in oxalic acid solutions leads to pronounced changes in the CPD distribution, which reflect modifications of the Au surface work function resulting from electrochemical reactions and ion adsorption from the solution. According to the theory of CPD measurements, the observed signal arises from the difference in work functions between the measuring probe and the sample, which is directly related to their energy states and the presence of adsorbed or chemically modified surface layers formed during the electrochemical process [57].

The systematic decrease in the mean CPD value (CPD_av_) with increasing oxalic acid concentration indicates an increase in the effective work function of the gold surface. These changes result from the formation of surface dipoles during anodization and the adsorption of oxalate anions on the metal surface. Such modification leads to a change in the local charge distribution and a shift of the Fermi level toward more stable energy states, which is consistent with literature reports on the influence of adsorption on the work function of noble metals, including gold [29].

The literature clearly indicates that the CPD value strongly depends on the state of the metallic surface, including the presence of adsorbed molecules, ions, and chemical reactions occurring at the surface. These factors affect charge distribution, surface dipole formation, and thus the effective work function of the material [29,57]. In the case of gold, changes in the work function induced by the adsorption of foreign chemical species (atoms, molecules, or ions) are a well-documented mechanism responsible for the observed variations in the contact potential [29].

The observed small changes in the skewness of the CPD distributions (CPD_sk_ values close to zero) suggest that the chemical modification of the surface proceeds in a relatively spatially homogeneous manner. This implies that the gold surface is covered by a layer with a similar degree of modification, without pronounced macroscopic heterogeneities within a single sample. At the same time, positive kurtosis values (CPD_ku_) and their increase with acid concentration indicate the emergence of more pronounced local CPD fluctuations. This phenomenon can be associated with more intense anion adsorption and local microstructural changes of the Au surface at higher electrolyte concentrations [29,57].

In an electrochemical context, the observed changes in CPD and the metal work function after anodization may also be related to the formation of thin oxide or hydroxylated layers, as well as to changes in the structure of the electrical double layer at the electrode–electrolyte interface. Such effects are characteristic of metal surface oxidation processes in acidic environments and have been widely described in the electrochemical and surface physics literature. From this perspective, CPD serves as a sensitive indicator of chemical and electronic changes occurring at the metal surface as a result of adsorption and electrochemical reactions [29].

The CPD measured by SKP reflects changes in the effective work function of anodized gold surfaces; however, this relationship is not strictly linear and should be interpreted as a surface-averaged response. The observed CPD variations arise from the synergistic contribution of nanostructure geometry and surface chemistry. From a geometric perspective, increased surface curvature, ligament-based morphology, and a higher density of low-coordinated surface atoms modify the local electrostatic potential and electron spill-out at the metal surface. Simultaneously, surface chemistry effects, including adsorption of oxalate-derived species and the formation of surface dipoles, further alter the effective work function. As a result, CPD represents an averaged parameter that is highly sensitive to local electrostatic potential fluctuations rather than a direct measure of a single intrinsic material property [1,49].

### 3.4. The Effect of Anodizing on the In Vitro Corrosion Resistance of p-Au

**Figure 7:**
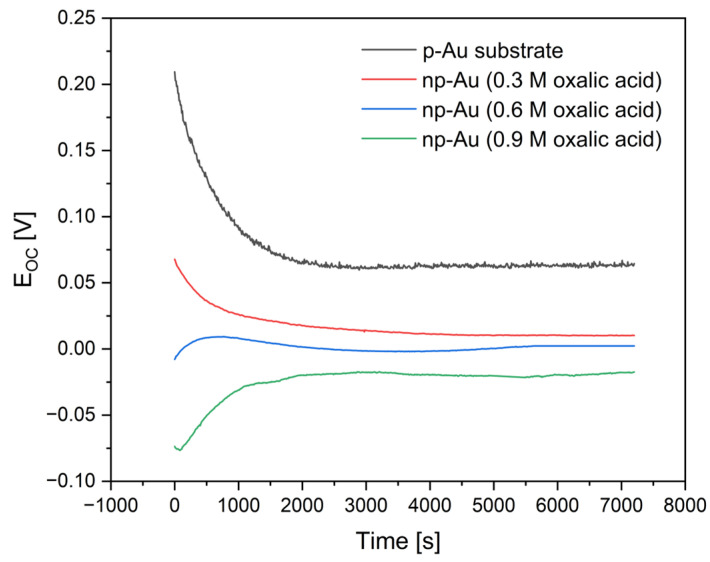
Open circuit potential (E_OC_) vs. time in artificial saliva for polycrystalline gold (p-Au) and nanoporous gold (np-Au) obtained by anodization in oxalic acid at concentrations of 0.3 M, 0.6 M, and 0.9 M.

Open circuit potential measurements as a function of time performed in artificial saliva revealed significant differences in the electrochemical behavior of p-Au and np-Au obtained by anodization in oxalic acid at different concentrations (Figure 7).

The highest and consistently positive E_OC_ values over the entire measurement period were exhibited by the p-Au electrode. The initially recorded potential was approximately 0.21 V and gradually decreased, stabilizing at around 0.064 V, indicating rapid attainment of electrochemical equilibrium and high thermodynamic stability of the material in the investigated environment. Such behavior is characteristic of smooth and homogeneous surfaces of noble metals and reflects a low susceptibility to surface reactions in artificial saliva. In the case of np-Au, a clear decrease in E_OC_ values compared to p-Au was observed, with the effect becoming more pronounced as the concentration of oxalic acid used during anodization increased. The np-Au samples anodized in 0.3 M oxalic acid exhibited positive E_OC_ values that gradually stabilized at approximately 0.01 V. The lower potential relative to p-Au can be attributed to the significantly higher specific surface area and the presence of numerous structural defects, which enhance the electrochemical reactivity of the material. Further increasing the concentration of the anodizing electrolyte to 0.6 M resulted in a shift of the E_OC_ toward values close to zero. The potential was initially slightly negative and subsequently stabilized near 0 V over time. This behavior indicates a state of borderline electrochemical stability, arising from a more developed porous structure and stronger interactions between the np-Au surface and the components of artificial saliva. The lowest and most negative E_OC_ values were recorded for np-Au anodized in 0.9 M oxalic acid. The potential stabilized at approximately −0.017 V, reflecting the highest electrochemical reactivity among the investigated samples. This pronounced negative shift in E_OC_ can be associated with the maximum development of the nanoporous structure, a high density of active sites, and an increased capacity for adsorption of ions present in the electrolyte environment.

The obtained results clearly demonstrate that an increase in the degree of gold nanoporousness, controlled by the oxalic acid concentration during anodization, leads to a systematic decrease in the open circuit potential in artificial saliva. This trend indicates a reduction in the thermodynamic electrochemical stability of the material, although gold retains its noble metal character. The electrochemical stability of the investigated samples decreases in the following order: p-Au > np-Au (0.3 M) > np-Au (0.6 M) > np-Au (0.9 M). These findings highlight the crucial influence of surface morphology on the electrochemical behavior of gold in environments simulating oral conditions.

**Figure 8:**
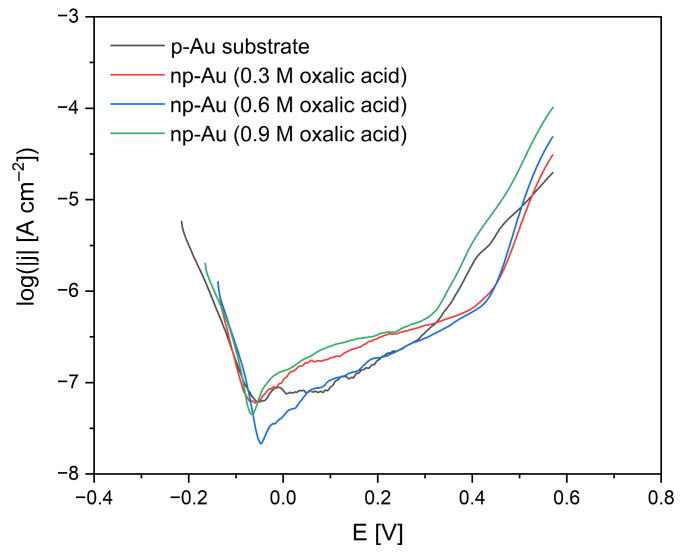
Anodic polarization curves recorded in artificial saliva for polycrystalline gold (p-Au) and nanoporous gold (np-Au) obtained by anodization in oxalic acid at concentrations of 0.3 M, 0.6 M, and 0.9 M, illustrating the effect of anodization conditions on electrochemical stability and anodic behavior.

Anodic polarization curves recorded in an artificial saliva solution for p-Au prior to anodization and for np-Au obtained by anodization in oxalic acid at concentrations of 0.3 M, 0.6 M, and 0.9 M provide important information on the electrochemical stability of these materials under conditions close to the oral environment (Figure 8).

The p-Au electrode exhibits the typical behavior of a noble metal with high corrosion resistance in physiological media, which is reflected in low anodic current densities maintained in the range of log j ≈ −5.3 to −5.4 A·cm^−2^ and the absence of a sharp increase in current within the investigated potential range. The corrosion potential of p-Au, approximately −0.21 V, is consistent with values reported for gold in saliva-simulating solutions, while the onset of anodic processes observed at potentials of about 0.25 to 0.30 V can be attributed to the formation of a thin and stable Au(I)/Au(III) oxide layer that effectively limits further oxidation of the metal [58]. Such behavior confirms the high biostability of gold used in dentistry, while at the same time indicating its limited surface activity.

The introduction of a nanoporous structure as a result of anodization leads to pronounced changes in the anodic polarization curves, which are directly related to the significant increase in the real electrochemical surface area and the presence of numerous structural defects. For np-Au anodized in 0.3 M H_2_C_2_O_4_, a decrease in current density in the low-potential region to values of about log j ≈ −6.0 to −6.1 A cm^−2^ is observed, along with a gradual increase in current with increasing potential, a corrosion potential of approximately −0.14 V, and an anodic process initiation potential of 0.20 to 0.25 V. With increasing oxalic acid concentration to 0.6 M and 0.9 M, a systematic increase in anodic current density is observed, accompanied by a lowering of the anodic process initiation potential to approximately 0.15 to 0.20 V and 0.10 to 0.15 V, respectively, as well as an increased contribution of active processes associated with unstable gold oxide formation and local surface degradation. These phenomena are characteristic of highly developed nanoporous structures, which exhibit high electrochemical activity but reduced corrosion resistance in biological environments [34]. Consequently, although the highest concentration of the anodizing electrolyte leads to the greatest surface activity, the most favorable compromise between electrochemical stability and bioactivity in an artificial saliva environment is offered by np-Au anodized in 0.3 M H_2_C_2_O_4_, making this material the most promising for long-term dental applications.

Analogous to the observed influence of anodization conditions on the onset potential and anodic activity of np-Au, it was reported that modifications to artificial saliva using mouthwashes or fluoride additives significantly altered the corrosion kinetics and passive film characteristics of dental materials, demonstrating that both surface structure and environmental chemistry critically affect electrochemical behavior [59].

It should be emphasized that in the context of this work, the term ‘biomedical and dental applications’ refers to the application potential of anodized np-Au surfaces arising from the well-established biocompatibility of gold combined with their tailored surface morphology, electronic properties, and electrochemical behavior. A comprehensive biological evaluation, including cell adhesion, proliferation, and cytotoxicity studies, represents an important subsequent research step but lies beyond the scope of the present study.

## 4. Conclusions

Electrochemical anodization of p-Au in oxalic acid solutions enables effective and controllable surface nanoengineering, resulting in the formation of np-Au layers with tunable structural, electronic, and electrochemical properties. Increasing the concentration of oxalic acid leads to systematic growth of the nanoporous layer thickness, enlargement of pore size, and increased surface porosity, accompanied by greater morphological heterogeneity at high concentrations. Surface-sensitive chemical analysis confirmed the formation of oxygen-containing surface species after anodization, while gold remained the principal component of the material.

Anodization induces pronounced changes in the electronic properties of gold, as evidenced by CPD measurements, which reflect modifications of the surface work function associated with nanostructure development and anion adsorption. Corrosion studies in artificial saliva demonstrated that increasing nanoporousness reduces the thermodynamic electrochemical stability of gold; however, the material retains its noble character. Among the investigated conditions, anodization in 0.3 M oxalic acid provides the most advantageous compromise between structural uniformity, electronic homogeneity, and corrosion resistance.

The results demonstrate that moderate oxalic acid anodization (0.3 M) provides an optimal np-Au surface in which the effective SSA, electronic homogeneity, and corrosion resistance are simultaneously maximized, while further increases in porosity lead to structural coarsening and diminished functional performance. They confirm that oxalic acid anodization is a simple, scalable, and chemically mild method for producing functional np-Au surfaces. The ability to tune surface morphology and properties by adjusting anodization parameters makes this approach particularly promising for dental and biomedical applications where controlled surface activity and long-term stability are required.

## Figures and Tables

**Table 1:** Chemical composition of AFNOR artificial saliva solution [47].

Component	Content [g·L^−3^]
NaCl	6.70
KCl	1.20
Na_2_HPO_4_	0.26
KH_2_PO_4_	0.20
NaHCO_3_	1.50
KSCN	0.33

**Table 2:** Quantitative pore characteristics of nanoporous gold (np-Au) formed by anodization in oxalic acid solutions of varying concentrations, where SSA denotes the effective specific surface area of np-Au calculated using a geometric model based on SEM-derived pore size and surface porosity.

Oxalic Acid Concentration	Average Pore Diameter [nm]	Median Pore Diameter [nm]	Standard Deviation [nm]	Pore Density [1/µm^2^]	Surface Porosity [%]	Estimated SSA [m^2^/g]
0.3 M	25 ± 8	22	7	420	18	1.49
0.6 M	45 ± 15	42	13	280	32	1.47
0.9 M	85 ± 40	70	38	150	48	1.17

**Table 3:** CPD statistical parameters for the gold surface calculated using the results presented in Figure 5 and Figure 6.

Type of Sample	CPD_av_ [V]	CPD_q_ [V]	CPD_sk_	CPD_ku_
p-Au substrate	−0.1823 ± 0.0003	0.1829 ± 0.0003	−0.067 ± 0.061	0.534 ± 0.141
np-Au (0.3 M oxalic acid)	−0.2447 ± 0.0004	0.2454 ± 0.0004	−0.081 ± 0.080	0.662 ± 0.240
np-Au (0.6 M oxalic acid)	−0.2895 ± 0.0004	0.2900 ± 0.0004	−0.042 ± 0.074	0.746 ± 0.163
np-Au (0.9 M oxalic acid)	−0.3278 ± 0.0005	0.3286 ± 0.0005	−0.015 ± 0.069	0.812 ± 0.152

## Data Availability

The original contributions presented in this study are included in the article. Further inquiries can be directed to the corresponding authors.

## Abbreviations

The following abbreviations are used in this manuscript:

CPD	Contact potential difference
CPD_av_	Arithmetic mean of contact potential difference
CPD_ku_	Kurtosis of contact potential difference distribution
CPD_q_	Root mean square of contact potential difference
CPD_sk_	Skewness of contact potential difference distribution
CV	Cyclic voltammetry
EDS	Energy-dispersive spectroscopy
E_OC_	Open circuit potential
np-Au	Nanoporous gold
p-Au	Polycrystalline gold
SEM	Scanning electron microscopy
SKP	Scanning Kelvin probe
SSA	Specific surface area
TAS	Template-assisted synthesis
